# The use of arthrocentesis in patients with temporomandibular joint disc displacement without reduction

**DOI:** 10.1371/journal.pone.0212307

**Published:** 2019-02-13

**Authors:** Eduardo Grossmann, Rodrigo Lorenzi Poluha, Lilian Cristina Vessoni Iwaki, Rosângela Getirana Santana, Liogi Iwaki Filho

**Affiliations:** 1 Department of Dentistry, Federal University of Rio Grande do Sul, Porto Alegre, Rio Grande do Sul, Brazil; 2 Department of Dentistry, State University of Maringá, Maringá, Paraná, Brazil; 3 Department of Statistics, State University of Maringá, Maringá, Paraná, Brazil; Eberhard-Karls-Universitat Tubingen Medizinische Fakultat, GERMANY

## Abstract

The aim of this study was to evaluate the efficacy of the use of the arthrocentesis in patients with disc displacement without reduction (DDWOR). Two hundred and thirty-four (234) patients with DDWOR were evaluated and the following data collected: gender; affected side; age (years); duration of the pain (months); patient's perception of pain (measured by Visual Analogue Scale [VAS 0–10]); maximal interincisal distance (MID) (mm); and joint disc position, determined by magnetic resonance imaging. Data were obtained in two different moments: before the arthrocentesis (M1) and three or four months later (M2). Paired t-Student Test, Scores Test and Wilcoxon Test showed a statistical significant difference (p<0.0001) between the M1 and M2 for the variables VAS and MID. There was an alteration in the joint disc position in 93.88% of the cases after arthrocentesis. There was no association between the general characteristics of the patients on the M1 and the results of the arthrocentesis (p>0.05). It can be concluded that the arthrocentesis is efficient in reducing the pain, in increasing interincisal distance, and altering the joint disc position in patients with DDWOR regardless gender, age side and pain duration.

## Introduction

Among the different kinds of temporomandibular joint (TMJ) disorders, the displacement of the disc without reduction (DDWOR) has a prevalence of 35.7% [1]. In this condition, either with the mouth open or closed, the disc remains anteriorly displaced in relation to the condyle, being pain and mouth opening limitation the main clinical features [2–4]. The treatment for DDWOR should, at first, be a reversible and conservative one (drugs, interocclusal devices and physiotherapy) [5], however if these methods are not efficient, surgical procedures should be considered [6].

Arthrocentesis is a minimally invasive joint surgery and it is effective for decreasing pain, increasing maximal interincisal distance, eliminating joint effusion and improving the oral health related to the quality of life of the patients with TMJ disorders [6, 7]. It consists in washing the superior compartment of the TMJ, which is performed without a direct visualization of the performance. The washing procedure is done using a biocompatible substance, such as a physiological solution, which dilutes the local algogenic substances and frees the joint disc by removing the adhesions formed between the surfaces of it and the mandibular fossa, which is free due to the hydraulic pressure generated by the irrigation process [6, 8–11].

The arthrocentesis technique was introduced at about 30 years ago [8] and it has been largely used along with other treatments, such as infiltrations of sodium hyaluronate [12], intra-articular analgesics [13], corticosteroids [14] and platelet rich plasm [15]. However, the literature has showed that an investigation regarding the isolated effects of the arthrocentesis over the DDWOR along with a clinical analysis performed with the aid of the Magnetic Resonance Imaging (MRI) may elucidate some of the benefits of this therapy.

Therefore, the aim of this study is to evaluate the efficacy of the arthrocentesis, alone, in patients with DDWOR. The null hypothesis to be tested says that the results of the variables studied here will not show any difference before and after the arthrocentesis.

## Materials and methods

The research was approved by the Ethics Committee for Research on Humans of the State University of Maringá (N°: 1.751.299). A retrospective observational study was conducted based on the Helsinki Declaration and recommendations of the report entitled Strengthening the Reporting of Observational Studies in Epidemiology (STROBE) [16]. Data were collected from the medical records of 400 patients in an orofacial pain and deformity centre (CENDDOR) in Porto Alegre, Brazil, between January 2006 and May 2018. Clinical examinations and procedures were conducted by the same surgeon (E.G.).

The patients included in the study should be 18 years old or above, have clinical symptoms compatible with DDWOR and joint pain that did not respond to a conservative treatment for at least three months (occlusal splints, anti-inflammatory drugs, compresses, soft diet and physiotherapy). The diagnosis of DDWOR were confirmed by a combination of a clinical examination based on the axis I of the Research Diagnostic Criteria for Temporomandibular Disorders (RDC/TMD) [2] and by MRI reports.

About the initial 400 patients, 20 had incomplete medical records were excluded; 59 present rheumatoid arthritis; 2 presented agenesis, 2 hyperplasia, 2 hypoplasia e 1 had a malignant neoplasm in the condyle; 5 presented bone ankylosis; 15 had previous TMJ surgery; 10 reported extreme fear for needles; and 50 no performed a MRI before the arthrocentesis and were also excluded from the sample. A total of 234 patients (245 TMJs) fit into the research criteria.

The following variables were registered: gender; side affected by joint pain; age (years); pain duration (months); patient’s pain perception (measured by Visual Analogue Scale–VAS (0–10)); maximal interincisal distance (MID) (mm), measured by a digital calliper (Mitutoyo, Takatsu-ku, Kawasaki, Kanagawa, Japan); and joint disc position, determined by MRI. The data were obtained in two different moments: before the arthrocentesis (M1) and three to four months later (M2).

### Magnetic resonance images

MRI scans were obtained in a Magnetic Resonance device at 1.5 Tesla (Signa HDxt; GE Healthcare, Milwaukee, WI, USA). Series of T1 weighted images were performed with a repetition time (RT) of 567 milliseconds and time echo (TE) of 11.4 milliseconds. The Series of T2 weighted images were performed with a RT of 5.200 milliseconds and a TE of 168.5, with bilateral spherical surface coil of 9cm diameter. The matrix used for T1 was 288 x 192, with numbers of excitation NEX = 3; and for T2, 288 x 160, with NEX = 4; and with a field of view (FOV) of 11x11 cm.

The MRI images were all analysed by the same radiologist, who based his analysis on the studies of Ahmad et al. (2009) [3]. The reports obtained in the second MRI were used to classify the final position of the articular disc in relation to the first MRI, in three categories: No change (NC); the disc remained in the initial position, but there was anterior and inferior movement of the condyle during mouth opening (IPAIC); and, there was a more anterior and inferior movement of the set of the condyle/disc in relation to the articular tubercle (MAICD).

### Arthrocentesis

**Fig 1:**
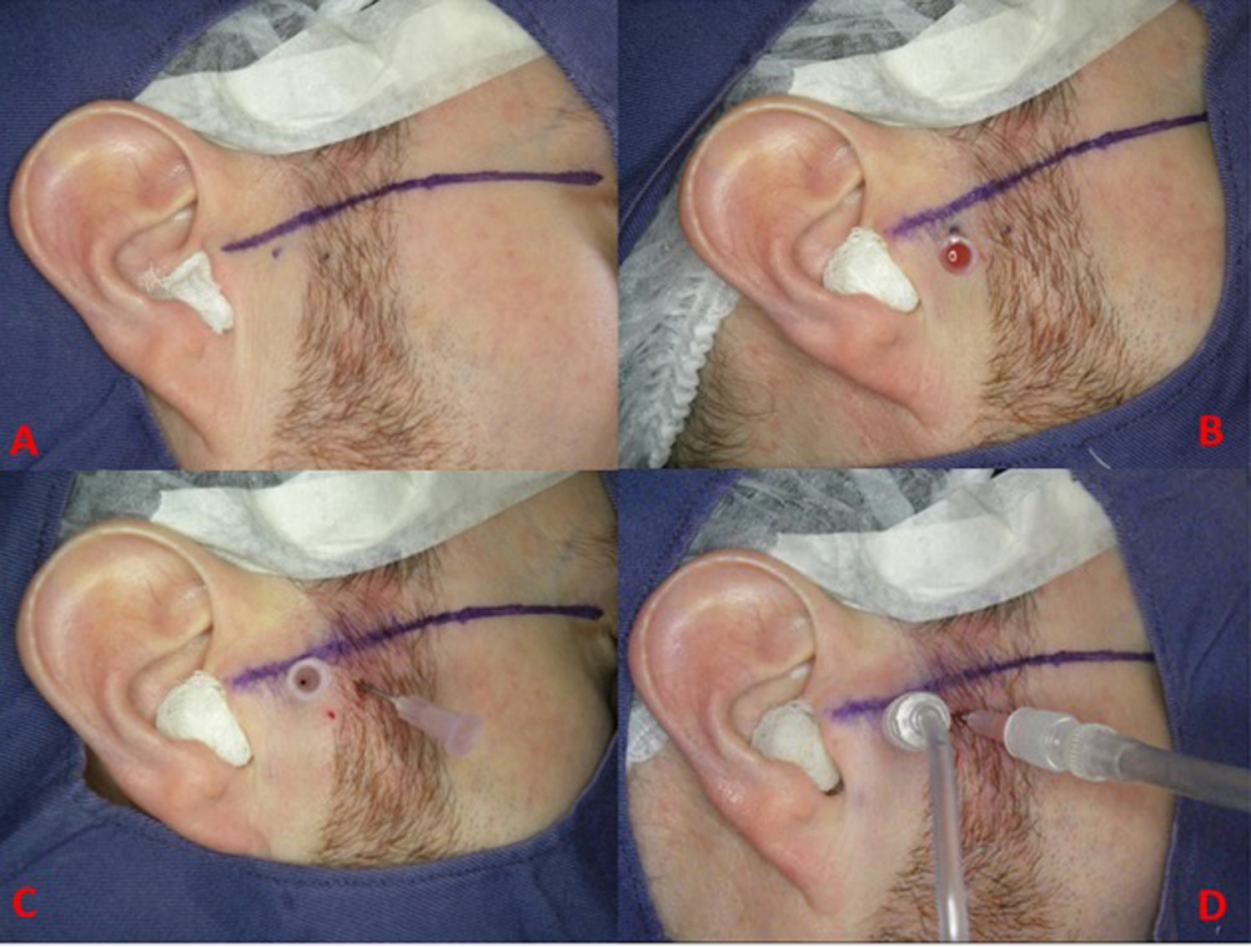
A: reference line and the two points for the insertion of the needles. B: first needle inserted. C: second needle inserted. D: first needle connected with syringe and the second needle connected to a vacuum pump. The physiological solution is administered in the first needle, get into the superior compartment of TMJ and got out by the second needle.

The Arthrocentesis was performed once only in each of the indicated joint and the procedure followed the technical references found in the literature [6,8, 10–15, 17] (Fig 1). A demographic pen was used to draw a straight line from the middle portion of the tragus to the corner side of the eyeball and two points were marked on this line for the insertion of the needles: the first, the most posterior one, at a distance of 10 mm from the tragus and 2 mm below the corner-tragus line; the second was inserted 20 mm anterior to the tragus and 10 mm inferior to the corner-tragus line. Antisepsis was performed with 2% chlorhexidine solution that was used all over the face, mainly in the preauricular area and ear. The next step was the auriculotemporal nerve block, followed by the anaesthesia of the posterior deep temporal and masseter nerves with lidocaine chloride without vasoconstrictor (Xylestesin 2%. Cristália, São Paulo, São Paulo, Brazil), with a total volume of 3.6 mL. The patient was then requested to open the mouth to the maximum, and a sterile mouth opener was placed on the contralateral side of the procedure, allowing the displacement, down and forward, of the condyle, which enabled the access to the posterior recess of the superior compartment of the temporomandibular joint where the first needle 40x12 mm (18G) was introduced. The needle was then connected with a 5mL syringe and 4 mL physiological solution (PS), sodium chloride 0.9%, was administered in order to distend the joint space. A second needle was introduced into the distended compartment, at the point established before, and connected to a No° 20 long (60 cm) flexible and transparent catheter connected to a vacuum pump (KaVo Vacuum, Kavo, Joinville, Santa Catarina, Brazil), which allowed the visualization of the solution. Afterwards, an infusion extender, 15C of 120 cm (Compojet Biomedica, Conceição do Jacuípe, Bahia, Brazil), was connected to a 60 mL syringe to allow the joint lysis and lavage. A total of 300 mL of physiological solution was used for the TMJ arthrocentesis. No other substance or drug was added to the solution being injected. Once the procedure was completed, the needles were removed. Local dressing was conducted with sterile gauze and micropore. After the procedure, the patients were not recommended to use the occlusal splints or realized any other treatment, during the follow-up period.

### Statistical analysis

The individuals were considered as observational units; data were tabulated and submitted to a descriptive analysis. The variables, VAS and MID, were evaluated before and after arthrocentesis (M1 and M2) and the results analysed by Paired Wilcoxon Test. In addition, univariate analysis by logistic regression model was used to check if the characteristics of the patients collected in the M1 (gender; affected side; age; pain duration) were responsible for the effects of the arthrocentesis on the variables of interest (VAS and MID). All tests were performed with a significance level of 5%. Data were analyzed using SAS version 9.3 (SAS Institute Inc., Cary, NC, USA).

## Results

The descriptive variables are shown in Table 1. The averages, standard deviation (±SD), minimum, medium and maximum values of the VAS and MID variables are in Table 2.

**Table 1:** Distribution of the frequency, average and standard deviation (±SD) of the descriptive variables.

		Female	Male	Total Sample
**Gender**		208 (88.88%)	26 (11.12%)	234 (100%)
**Side of the complaint**	**Right**	110 (52.88%)	10 (38.46%)	120 (51.28%)
	**Left**	89 (42.78%)	14 (53.84%)	103 (44.01%)
	**Both**	9 (4.34%)	2 (7.70%)	11 (4.71%)
**Age (years)**		32.84±8.19	33.21±7.49	33.02±7.84
**Pain Duration (months)**		10.40±4.85	11.70±4.86	11.05±4.85

**Table 2:** Descriptive measures of the variables VAS and MID.

	Measures	VAS (0–10)	MID (mm)
**M1**	**Average ±SD**	7.20±1.37	31.04±1.68
	**Minimum**	5	25.17
	**Medium**	8	30.49
	**Maximum**	10	35.00
**M2**	**Average ±SD**	0.43±0.44	42.50±4.09
	**Minimum**	0	37.14
	**Medium**	0	45.22
	**Maximum**	1	54.05

VAS: visual analogue scale. MID: maximal interincisal distance. M1: before the arthrocentesis. M2: three or four months after arthrocentesis.

The paired Wilcoxon test showed statistically significant differences (p < 0.0001) between M1, before, and M2, after the arthrocentesis, for VAS and MID. The univariate analysis by logistic regression model showed no association between the characteristics of the patients collected in the M1 (gender; affected side; age; pain duration) with the results of VAS and MID after the arthrocentesis (p>0.05).

**Fig 2:**
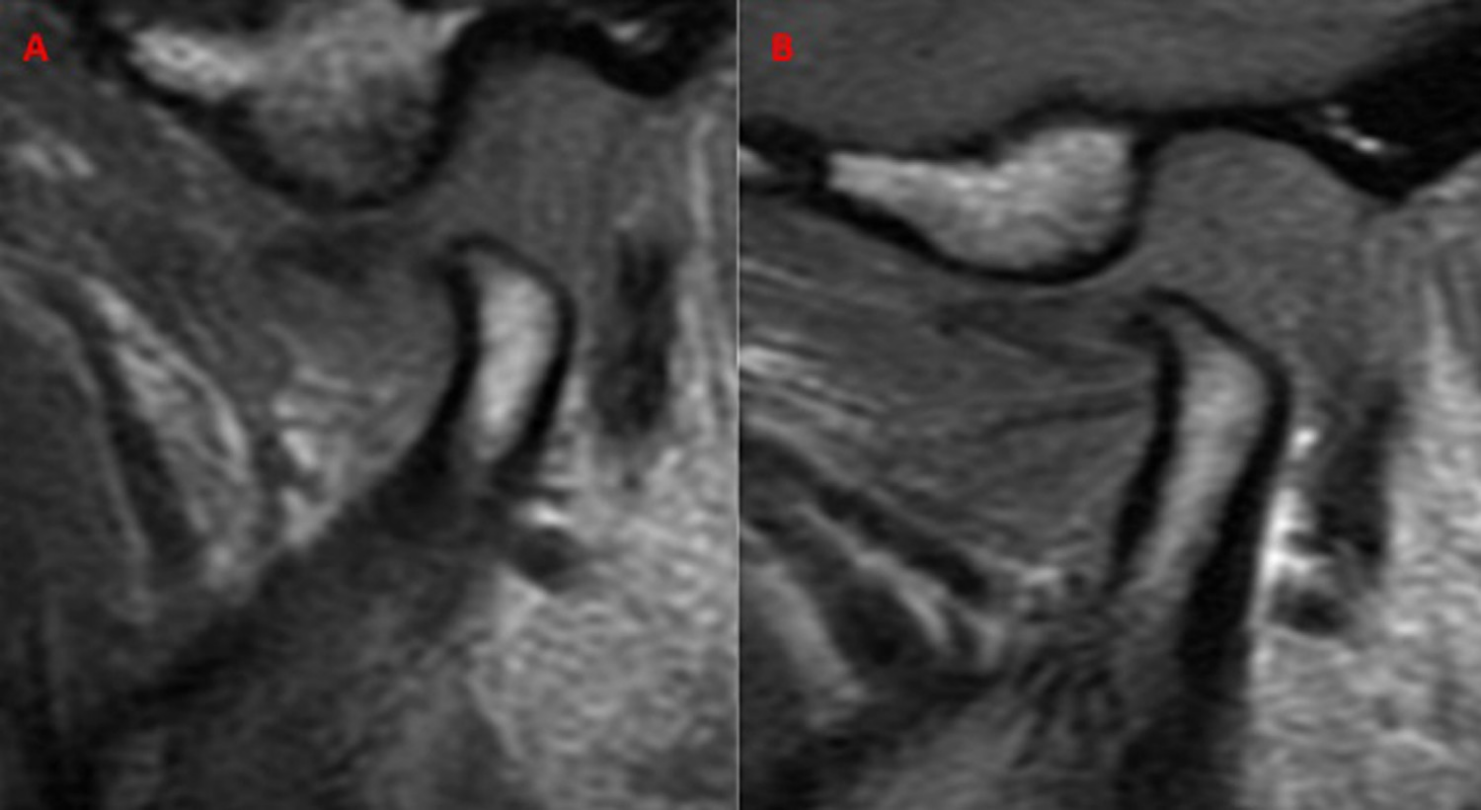
NC example. A: before the arthrocentesis. B: four months after arthrocentesis.

**Fig 3:**
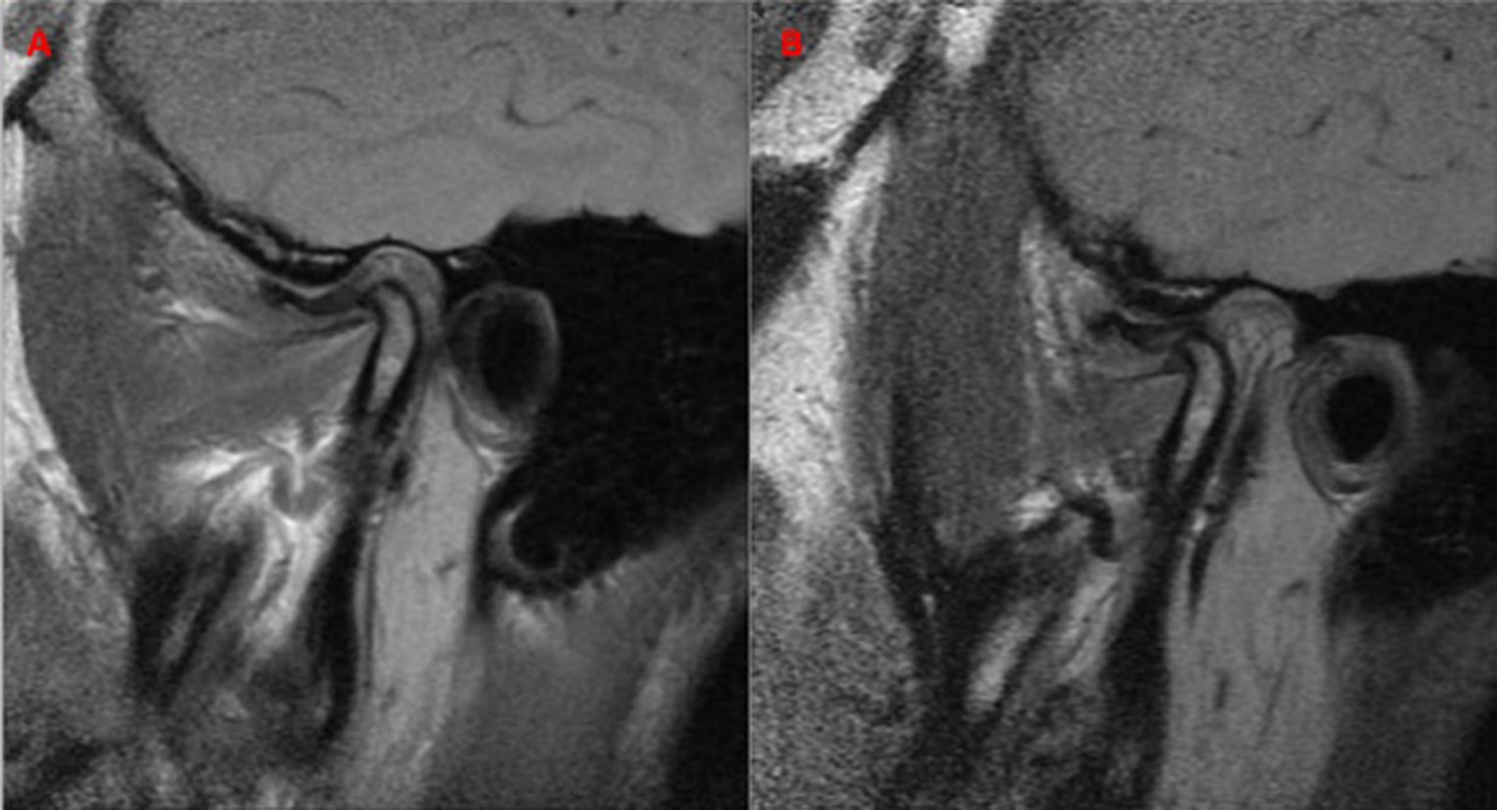
IPAIC example. A: before the arthrocentesis. B: four months after arthrocentesis.

**Fig 4:**
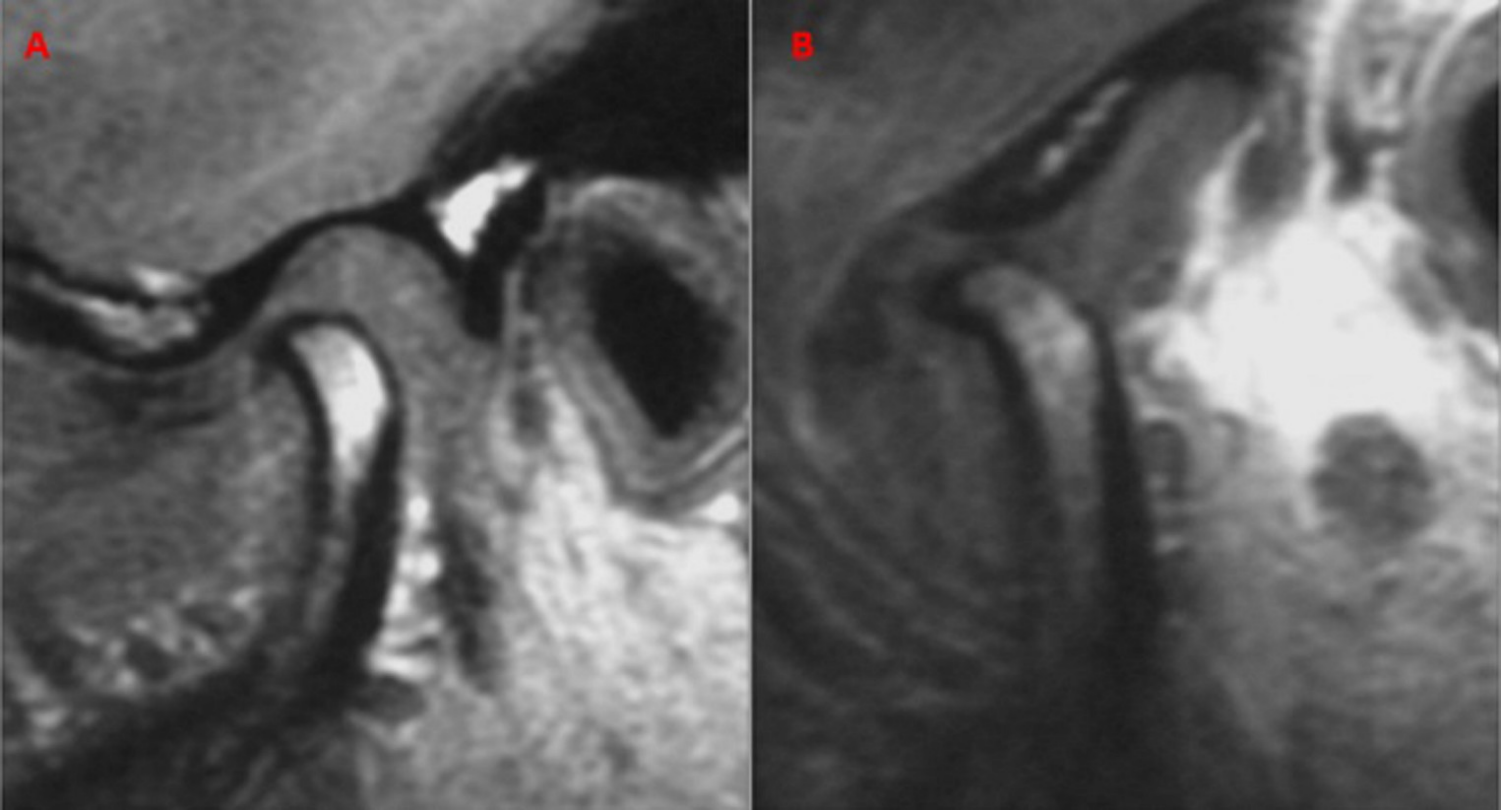
MAICD example. A: before the arthrocentesis. B: four months after arthrocentesis.

The results of the second MRI revealed that there was no change (NC, Fig 2) in 6.12% of the cases; in 14.69% of the patients, the disc remained in the initial position, but there was an anterior and inferior movement of the condyle during mouth opening (IPAIC, Fig 3); and in 79.19% of the cases there was a previous and inferior movement of the set of the condyle/disc in relation to the articular tubercle (MAICD, Fig 4), suggesting a tendency towards this position (Table 3).

**Table 3:** Distribution of the position of the TMJ disc according to the second MRI.

Position of the joint disc–MRI	Female (217 TMJs)	Male (28 TMJs)	Total (245 TMJs)
**NC**	13 (6%)	2 (7.14%)	15 (6.12%)
**IPAIC**	30 (13.82%)	6 (21.42%)	36 (14.69%)
**MAICD**	174 (80.18%)	20 (71.44%)	194 (79.19%)

NC: no change. IPAIC: the disc remained in the initial position, but there was anterior and inferior movement of the condyle during mouth opening. MAICD: there was a more anterior and inferior movement of the set of the condyle/disc in relation to the articular tubercle.

## Discussion

Although most DDWOR patients are asymptomatic, some individuals can experience pain and reduced jaw mobility [18], being the arthrocentesis an effective treatment option for DDWOR when conservative methods are no longer efficient [10]. The results of the present study showed that DDWOR patients who underwent arthrocentesis had their VAS reduced and MID increased (p<0.0001), which gave us support to reject the null hypothesis.

Pain is the most recurrent reason for patients to seek treatment for their TMJ [4], and the earlier the patient undergoes treatment, the better the results are. Lately, arthrocentesis has seemed to be more effective in treating pain [5], and some studies have also showed that its cost-effectiveness has been superior to the more conservative approaches [19]. In the present study, there was a significant reduction (p<0.0001) in the perception of pain after arthrocentesis, with VAS values reducing from 7.20±1.37 to 0.43±0.44, being these values similar to the ones found in other studies, from 8.7±1.1 to 1.13±1.16 [6] and from 6.45±1.17 to 1.12±0.42 [20].

The reduction of the pain is expected as the irrigation process, conducted with bio-compatible substances, allows the removal of debris of the joint tissues in degeneration and eliminates allogeneic substances, mainly inflammatory mediators [7, 10, 21]. The arthrocentesis is considered to be effective when there is a reduction of the levels of these mediators [22], being the proper use of this technique highly important to achieve good results. In this study, was used 300 mL of physiological solution; however, recent literature has showed that smaller volumes (50–200 mL) are equally effective to wash the upper compartment of the TMJ [23].

Despite reducing pain, the arthrocentesis performed under pressure may also present other benefits to the patient, as it removes adherences, eliminates the negative pressure in the joint, distends the joint space, recovering the space of the joint disc and fossa, changes the viscosity of the synovial liquid, helps in the translation of the joint disc and condyle, and, consequently, enlarges mouth opening [8, 24–27]. After arthrocentesis, our patients experienced an increase in the maximum mouth opening, from 31.04±1.68 mm to 42.50±4.09 mm (p<0.0001), similar to what has been found in other studies (from 23.7 ±2.91 mm to 41.05 ±2.91 mm) [20] and (from 32.13±9.86 mm to 46.6±2.56) [6].

The proportion men: women were of 8:1, similar to the ones previously found in the literature [13, 28]. The higher incidence of DDWOR in women may be explained by some female characteristics, such as a higher muscle laxity, higher intra-joint pressure and hormonal changes [1, 29]. Regarding the side of the complaint, 95.29% of the patients presented unilateral complaints, and 4.71% bilateral [1]. In agreement with previous studies [21, 23], the present results showed no significant association between gender and the side of the joint in pain with VAS and MID results (p>0.05). The average age of the patients was of 33.02±7.84, also similar to the ones found in the literature [30]. However, there was no significant association between the age and VAS and MID (p>0.05), as it would be predicted, as the literature has showed that the rate of success of the arthrocentesis decreases as the age increases [31, 32]. Cases of DDWOR may be considered acute when the symptoms last for at least four months [33]. In the present sample, the average time reported for pain duration was of 11.05±4.85 months and there was no association between pain duration and VAS and MID; however, the literature has suggested that the arthrocentesis has more chance of being successful when DDWOR patients have not been in pain for long [31].

Within time, the displaced joint disc becomes significantly deformed, and the retrodiscal tissue becomes less flexible and more fibrous, which does not allow it to reposition itself along the condyle, both in open and closed mouth [18, 22]. Our data showed an alteration in the joint disc position in 93.88% of the cases after arthrocentesis (Table 3). From those, in 14.69% of the patients the disc remained in the initial position, but there was an anterior and inferior movement of the condyle during mouth opening; and in 79.19% of the cases there was a more anterior and inferior movement of the set of the condyle/disc in relation to the articular tubercle, being these changes probably due to combination of the following factors: distension of the upper compartment, joint washing under certain pressure and joint manipulation. The change in the position of the disc in DDWOR patients after arthrocentesis can be observed in the techniques where one or two needles were used [34]. It was not possible to observe the full return of the disc to its correct position in our sample, both with the mouth open and closed. However, this is not necessary to relieve the pain and restore the proper function of the joint in patients with DDWOR [22, 27, 35], being the normalization of the functionality more important than the re-establishment of the joint anatomy.

The minimally invasive character of the arthrocentesis produces less post-operative morbidity if compared with other surgical techniques for the TMJ. The main disadvantages in relation to the arthroscopy is that: it is not possible to visualise the intra-joint pathology; there are limitations for performing biopsies; and it is more difficult to eliminate adherences or adhesions in more advanced stages [7, 10]. Although there was no complication associated to the arthrocentesis in this study, the literature has reported some risks, such as: extravasation of liquid to the surrounding tissue, lesion of the facial nerve, optical lesion, pre-auricular haematoma, arteriovenous fistula, trans articular perforation, intracranial perforation, extradural haematoma and intra-articular problems [36].

This study was conducted in only one place and within a restricted population, so the results should be carefully analysed, as there were some limitations. No control group was used, as all the patients suffered from pain and restrictions to open the mouth and a control group could have their disorders aggravated. Placebo was also not used due to ethical conflicts, as we would have to perform the anaesthetic blocks and insert the needles without injecting any substance. After the procedure, the patients were not recommended to use the occlusal splints or realized any other treatment, during the follow-up period, so that there would be no interference in the interpretation of the results. Although the follow-up period in the present study ranged from 3 to 4 for months, the results tend to be long-lasting, once the literature has shown good results for pain and jaw motion even after one year after the arthrocentesis [23].

Considering the results and the limitations of the present study, it can be concluded that the arthrocentesis is efficient in reducing the pain, in increasing interincisal distance, and altering the joint disc position in patients with DDWOR regardless gender, age side and pain duration.
